# Bacteriophage MS2 genomic RNA encodes an assembly instruction manual for its capsid

**DOI:** 10.1080/21597081.2016.1157666

**Published:** 2016-03-02

**Authors:** Peter G. Stockley, Simon J. White, Eric Dykeman, Iain Manfield, Ottar Rolfsson, Nikesh Patel, Richard Bingham, Amy Barker, Emma Wroblewski, Rebecca Chandler-Bostock, Eva U. Weiß, Neil A. Ranson, Roman Tuma, Reidun Twarock

**Affiliations:** a Astbury Center for Structural Molecular Biology, University of Leeds, Leeds, UK; b Department of Biology and Mathematics & York Center for Complex Systems Analysis, University of York, York, UK; c Center for Systems Biology, University of Iceland, University of Iceland Biomedical Center, Reykjavik, Iceland

**Keywords:** RNA bacteriophage, RNA packaging signals, virion assembly

## Abstract

Using RNA-coat protein crosslinking we have shown that the principal RNA recognition surface on the interior of infectious MS2 virions overlaps with the known peptides that bind the high affinity translational operator, TR, within the phage genome. The data also reveal the sequences of genomic fragments in contact with the coat protein shell. These show remarkable overlap with previous predictions based on the hypothesis that virion assembly is mediated by multiple sequences-specific contacts at RNA sites termed Packaging Signals (PSs). These PSs are variations on the TR stem-loop sequence and secondary structure. They act co-operatively to regulate the dominant assembly pathway and ensure cognate RNA encapsidation. In MS2, they also trigger conformational change in the dimeric capsomere creating the A/B quasi-conformer, 60 of which are needed to complete the *T*=3 capsid. This is the most compelling demonstration to date that this ssRNA virus, and by implications potentially very many of them, assemble via a PS-mediated assembly mechanism.

## Background

**Figure 1.**
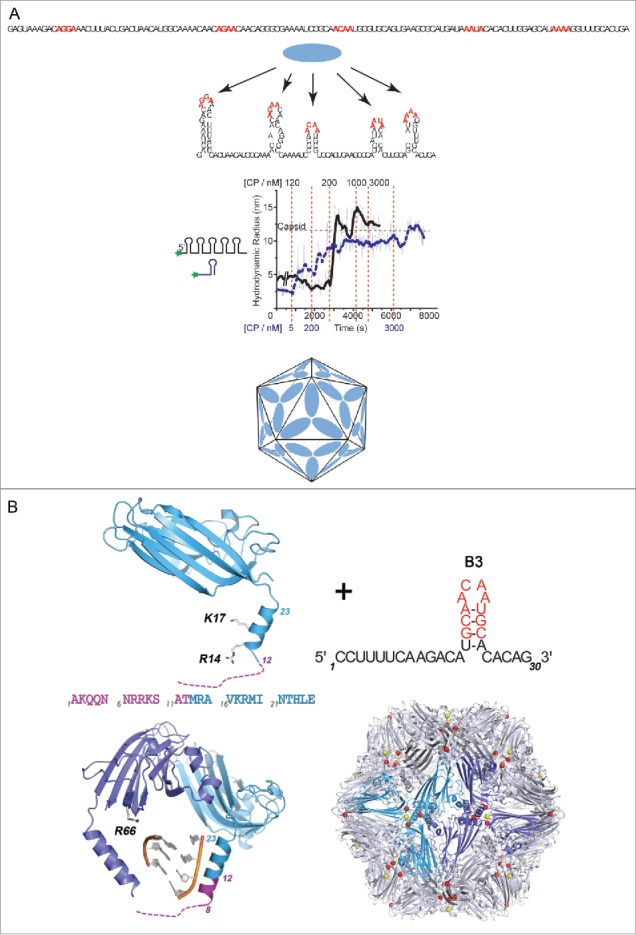
PS-mediated assembly of STNV. (A) Multiple, short, degenerate CP-recognition motifs within the STNV genome (highlighted in red) in the primary sequence (top), when presented appropriately by RNA folding into stem-loops, are bound co-operatively at high affinity (low nM) by cognate CPs. This is revealed by smFCS assays with oligos dye-labeled at one end (middle). An individual PS, shown to the left of the smFCS trace in blue, stimulates sequence-specific assembly when titrated with increasing CP concentrations. Titration points are shown below the trace also in blue. At a threshold concentration, ˜5 nM, the Rh shifts from around 3 nm to around 7 nm, consistent with formation of an RNA-CP capsomere, which we have shown contains 3 CP subunits. Thereafter, a *T* = 1 VLP is created, due to CP-CP interactions with this initiation complex that is only complete by 3 µM CP. In contrast, a viral fragment encompassing the first 127 nt at the 5′ end of the genome and predicted to form 5 PSs, reveals the co-operativity between these CP binding sites. The initial Rh is unchanged until the CP concentration reaches ˜120 nM, where it declines by about 20% mimicking effects we have seen on the full length genome (titration points shown in black above the trace). As CP concentration increases there is a rapid and complete transition to a *T* = 1 capsid, reflecting the co-operative interactions between CPs being mediated by the PSs. There are at least 2 stages of assembly, a rapid initial collapse of the genome preparing it for encapsidation into the limited space of its *T*=1 capsid (cartoon, bottom), and a slower assembly completion stage. (B) The molecular basis of PS action is revealed by the crystal structure of VLPs assembled around an RNA encompassing a high affinity PS, B3. In the presence of PSs, a region of the CP toward the N-terminus that is normally disordered forms an additional turn of α-helix. This region is rich in basic amino acids suggesting that PS binding overcomes an electrostatic barrier preventing CP-CP interaction from forming the trimeric capsomere.

**Figure 2.**
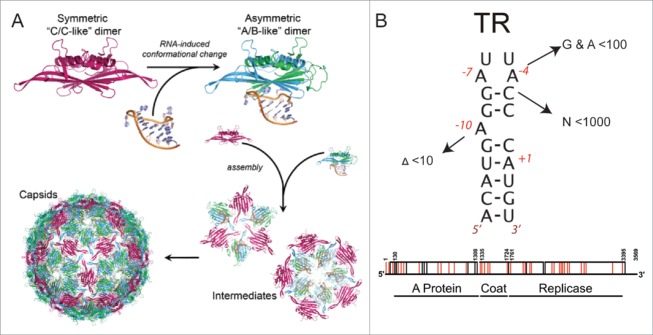
PS-mediated assembly of the *T* = 3 MS2 virion. (A) The capsomere in this case is a CP dimer, which in the absence of RNA is symmetrical in solution, consistent with it resembling the C/C quasi-dimer of the capsid. Binding to TR, and other related stem-loops, triggers a conformational change to the A/B-like dimer. Such RNA switching is required up to 60 times to make the *T* = 3 shell. (B) There is only a single copy of TR within the MS2 genome but many variant stem-loops can be formed that contain some of the important recognition features for CP^31^. Note, when these interactions (arrowed in figure) are measured *in vitro* their affinities are lowered significantly (shown as relative reduced affinities compared to TR). However, the concentration of a single stem-loop in the volume of the capsid is ˜0.3 mM. This compares with the K_d_ for TR binding to a CP dimer which is 1–10 nM, depending on the assays used. *In vivo* therefore many of the variant stem-loops can be expected to act as PSs. The locations of these variant PSs have been predicted and are shown below on the genetic map of the phage. Those that are also independently identified by the recent cross-linking within infectious phage are highlighted in red.

Single-stranded, positive-sense RNA phages co-assemble their protective coat protein shells around copies of their genomic RNA. This is one of the 2 common virus assembly mechanisms, the other being formation of a procapsid and the subsequent introduction of the viral genome, most commonly in the form of dsDNA, via specialized protein machinery. Spontaneous co-assembly is an essential feature of the lifecycles of a very wide range of important pathogens in every kingdom of life.^1^ Genome packaging in these viruses has been thought to be dominated by coat protein-coat protein (CP) interactions, and favorable electrostatic contacts that ensure RNA encapsidation, with at most a single, high-affinity CP-binding RNA signal.^2-6^ Recent work with a variety of viruses in this family suggests that these models are not representative of *in vivo* scenarios.^7^ When the first atomic structure of a spherical virus, namely that of Tomato Bushy Stunt Virus (TBSV), was revealed by Harrison and co-workers in a technical tour de force they defined the major structural questions that needed to be answered to explain that structure.^8^ These are how is genome encapsidation specificity achieved, and secondly how is the quasi-equivalence of the TBSV *T*=3 capsid established during assembly. We have now provided mechanistic explanations for both these events.^9-12^ We have shown that multiple RNA sites encompassing minimal CP sequence recognition motifs, when presented via RNA folding, regulate both packaging specificity and the pathway of virion assembly (Fig. 1). In effect, the RNA encodes a set of capsid assembly instructions, via multiple Packaging Signals (PSs). PSs facilitate formation of the protein-protein contacts in their capsids, and for bacteriophage MS2 it has been shown that they also act as allosteric switches determining the formation and placement of the quasi-conformers of the capsomeres forming the *T*=3 shell^13-17^ (Fig. 2).

*PSs facilitate virion assembly*: The plant satellite virus, Satellite Tobacco Necrosis Virus (STNV) also assembles using a PS-mediated mechanism. In that case the capsid is a *T* = 1 shell that encompasses a genome encoding only the CP gene, flanked by untranslated regions that enhance the efficiency of its replication and translation. STNV relies on a helper virus, Tobacco Necrosis Virus, for production of a replicase. In a *T* = 1 shell all the capsomeres, coat protein subunits, in the shell are in identical conformations. However, the STNV CP is similar to very many plant virus coat proteins, with a highly basic N-terminal region that is on the interior of the capsid and expected to interact with the RNA genome. Such electrostatic interactions were thought to provide the driving force for assembly, with the positively charged amino acids in the CP overcoming mutual repulsions between segments of the RNA chain. Using single molecule fluorescence correlation spectroscopy (smFCS) assays of STNV reassembly (Fig. 1) we have shown that this idea is incorrect. SmFCS allows us to monitor the hydrodynamic radius of dye-labeled species in real time at nanomolar (nM) concentrations, which are significantly lower than in most previous reassembly assays. When STNV RNA fragments are end-labeled with dye and then CPs are added under these conditions only cognate CPs generate an assembly reaction, and with the full length genome this results in a collapse of the hydrodynamic radius of the RNA preparing it for encapsidation. The collapse is clearly the result of multiple CP-RNA interactions. Such encapsidation specificity is characteristic of natural infections but is hard to rationalise via a purely electrostatic assembly mechanism. The CP alone does not self-assemble beyond monomer at low concentrations, up to micromolar, so these effects are RNA interaction dependent.

Multiple sites that could function as PSs were identified within the STNV genome using the technique of RNA SELEX directed against the CP, coupled to bioinformatic analysis of both sequence and secondary structure potential.^18^ They consist of RNA stem-loops displaying loops of 4, 5 or 6 nucleotides with the putative CP recognition sequence, -A.X.X.A-. These sites are distributed across both coding and non-coding regions of the genome. The first 125 nts at the 5′ end of the STNV genome are predicted to encompass 5 such PS sites both in the untranslated UTR and at the start of the CP gene. This fragment undergoes a collapse when STNV CP is added, followed by a co-operative assembly reaction forming the *T* = 1 shell. These reactions do not occur when the recognition motifs in all 5 stem-loops are replaced by –U.U.U.U-, confirming sequence specificity, and result in aberrant assemblies if the relative spacing of the stem-loops is altered. These results are consistent with a sequence-specific assembly initiation, leading to RNA collapse and subsequent VLP assembly. The PSs act by favoring the formation of the CP-CP contacts in the *T* = 1 shell and this effect is ablated by altered spacing of the wild-type PS elements. We have shown that these effects can be recreated in fragments that lack all the viral sequences except the CP recognition motif and the ability to form stem-loops at the same relative positions.

A structural explanation for this behavior comes from X-ray structure determination of VLPs assembled around multiple copies of single PSs at slightly higher concentrations^19^ (Fig. 1). This reveals that in the presence of the preferred RNA oligo the CP becomes more ordered at its N-terminus. In the virion, and VLPs assembled with *E.coli* RNAs, the basic N-terminal region is disordered below residue 12 with residues beyond this point forming an α helix. In the presence of the PS, order extends to residue 8 and an extra turn of α helix is formed. This peptide is very rich in basic amino acids which must cluster together around the particle 3-fold axes in a capsid. It appears that the PS placement on the CPs overcomes repulsive effects between CPs that prevent assembly of the CP alone.

### PSs also establish quasi-equivalent conformations

Bacteriophage MS2 is a *T* = 3 virion^20^ composed of 180 copies (actually 178 copies, see below) of an identical CP subunit organized as non-covalent dimers (Fig. 2). In contrast to the situation in STNV, MS2 must correctly form its 5-fold axes during assembly otherwise it will not be able to create a closed container for its 3569 nt genome. Structurally these symmetry axes are composed of an asymmetric quasi-conformer of the CP dimer, known as the A/B dimer, the B conformers forming the proteins surrounding the particle 5-fold axes. Symmetrical C/C dimers make up the rest of the capsid shell. All three CP conformers are distinguished by the conformation of a loop of polypeptide chain connecting the F & G β-strands of the CP fold. How each quasi-conformer is correctly placed is a major structural problem. We showed using non-covalent mass spectrometry,^13,15^ including ion mobility measurements,^17^ that the quasi-conformers could be switched by addition of oligonucleotides encompassing the TR sequence, the RNA-CP interaction favoring formation of the A/B state, rather than C/C, even though the FG-loops are distal from the RNA binding site. Modeling demonstrated that other TR-like stem-loops, sharing elements of the TR recognition motif for the CP dimer, could trigger the same allosteric effect via a perturbation of the conformational dynamics of the protein favoring A/B,^14^ suggesting that other stem-loops in the genomic sequence could function similarly to TR. The RNA-free C/C dimer and the TR-bound A/B dimer are kinetically trapped intermediates *in vitro*, but rapidly form *T* = 3 VLPs in each other's presence, and indeed TR and TR-like stem-loops appear to be PSs in this system. This led to the idea that up to 60 stem-loops within the MS2 genome might be required to complete virion assembly.

### Latest work and its significance

The traditional approach to establishing the functionality of sequence/structures in biology is to mutate the region in question and examine how this alters the wild-type phenotype. This is a very powerful approach, but in the case of PS-mediated assembly cannot be attempted simply as the RNA sequence controls both its own folding and impacts on replicative and translational efficiency. However, in one approach^21^ the single copy TR site within the RNA was mutated so it would not fold into a stem-loop. Remarkably, this yielded phages that were wild-type when titred in *E.coli*. However, altering the TR recognition site in the CP, based on known crystal structures,^23-26^ was completely lethal. This shows that a single PS, even a high affinity one such as TR, is not by itself essential, as expected for an evolutionarily robust mechanism. The authors concluded that the genome contained other sites that could mimic TR.^22,31^ Motivated by the fact that other genomic stem-loops can also act as triggers of the required allosteric switch of the A/B dimers,^14^ we developed a novel combination of graph theory and bioinformatics^31^ to identify potential candidates for such additional PSs. As expected for PS-mediated assembly^27^, we identified an ensemble of PSs with a common recognition motif.

As an alternative to mutating all 60 potential PS sites and then trying to do controls for replication and translation, infectious virions have been interrogated to see if they show the expected features of PS-mediated assembly. Working with Cheng Kao's group at Indiana University, where they have developed the technology to interrogate RNA-protein contacts within virions using either formaldehyde or UV-cross-linking,^22,28-30^ we examined infectious MS2 virions to see if they showed evidence of PS-mediated assembly. The CP peptides in contact with the genome were determined by mass spectrometry, and the RNA oligos in contact with the CP shell by NextGen sequencing. The only CP peptides in contact with the RNA correspond to those known to form the TR binding site,^23-26^ consistent with repeated RNA-CP contacts acting as PS conformational switches^14^. The RNA fragments showed a remarkable correspondence with previous predictions^31,32^ of the non-TR PS sites based on structural data, SELEX, functional CP-binding assays and novel theoretical analysis tools developed by the group, and included the TR site that can be thought of as a positive control. The results are a completely independent confirmation of the PS-mediated assembly model.

**Figure 3.**
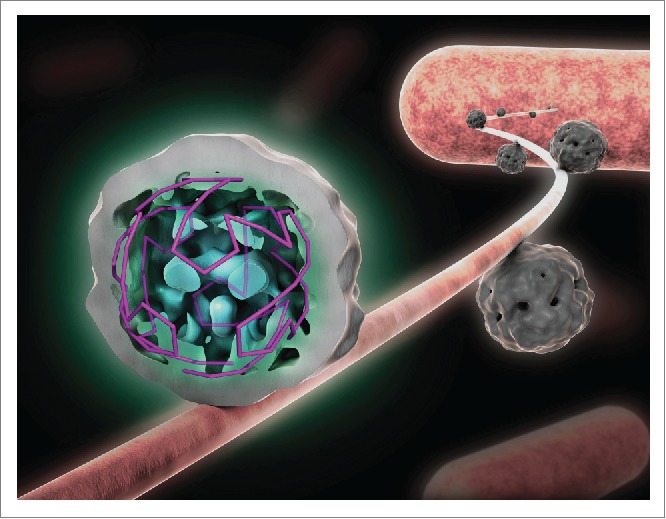
The implications of PS-mediated assembly for the earliest stages of infection. Shown is the asymmetric tomographic structure of MS2, at low resolution, bound to its initial cellular receptor, the *E.coli* F-pilus. This structure shows extensive density for the viral RNA, confirming the idea that PS-mediated assembly leads to almost identical RNA conformations within each viral particle. The contact to the pilus is mediated by the maturation protein, MP, which also binds to specific sites close to either end of the viral genome. Only the MP-RNA complex enters the bacterial cell, leaving the CP shell largely intact. MP replaces a C/C capsomere in an otherwise icosahedral protein shell.

Virions are necessarily transient structures, acting as transport vehicles for the genome between hosts. In a large number of cases the initial steps in infection for positive-sense, ssRNA viruses involve contact with a cellular receptor leading to conformational changes within the viral shell ultimately enabling the extrusion of the genome through a unique capsomer or vertex. For such an infection mechanism to occur the virus must be able to position one end of its RNA close to or beneath that special vertex. PS-mediated assembly can provide that control. In the case of MS2 there is an additional factor, namely a single copy maturation protein (MP) that both serves as the attachment point to the initial bacterial receptor, the pilus (Fig. 3) and guides the RNA-MP complex, formed via distinct MP PSs, into the target cell. Mathematical modeling of such an assembly reaction suggests that it ensures cognate and complete capsid assembly via the most stable assembly pathways,^11^ and that this can only be observed if the CP concentration is slowly ramped up as in a real infection. Perhaps this is one reason why PS-mediated assembly has been overlooked in previous *in vitro* reassembly reactions, while it was unambiguously demonstrated by us in the presence of such a ramp.^12^ In effect, the RNA forms a unique sequence of RNA-CP contacts with the overlying CP shell, a problem akin to the dilemma faced by a traveling salesman, i.e. identification of the best route to visit a number of defined sites once and only once while avoiding doubling back. These results imply an astonishing prediction, namely that there are only a very limited number of conformations of the RNA in proximity to the CP layer within every viral particle. Indeed, the interpretation of the recent cross-linking data assumes this to be the case.

This is a startling prediction, but there is independent evidence that this is true. An asymmetric tomographic^33^ reconstruction of the structure of MS2 bound to its initial cellular receptor, the bacterial pilus, although at low resolution, clearly reveals ordered density for the RNA (Fig. 3). Since this technique averages a large number of asymmetric objects, i.e., the individual virions bound to the pilus, this result can only occur if the RNA has the predicted similar conformation adjacent to the protein shell in different particles.^33^ This density can be analyzed in terms of the traveling salesman idea, and indeed corresponds to the layout expected from the assembly path based on the previously predicted and now experimentally validated PSs.^34^

The occurrence of such genome un-coating intermediates highlights the fact that all virions are asymmetric objects despite the highly symmetrical coat protein shells they assemble. The asymmetry comes in many forms: The genome is an asymmetric object, used in PS-mediated assembly to facilitate the construction of a symmetrical shell. PS-CP contacts formed impart asymmetric properties to each CP or capsomere contacted. Since PSs are sequence/structure degenerate their affinities for CP vary and hence they have differing impacts on CP dynamics. Another explicit form of asymmetry arises when the capsid shells contain unique structural features. For the RNA phages this comes in the form of a unique copy of a maturation protein (MP). The MS2 MP also has specific RNA binding sites on the viral genome located toward each end of the RNA, in effect making it circular and thus reducing the complexity of the potential conformations of the packaged genome, which has significant impact on virus assembly kinetics.^35^ Only the MP-RNA complex gets into the target bacterium with the CP shell remaining outside. The asymmetric tomogram^33^ suggests that the MP is an integral component of the external protein coat replacing one of the C/C quasi-conformers, hence the true CP content is 178 CPs, not 180. This may come as surprise to people used to looking at crystal structures or EM reconstructions of MS2 and the other RNA phages which are always portrayed as having wholly symmetrical icosahedral surface lattices of CPs. These are the result of using the icosahedral symmetry averaging to improve the electron density maps in each case. The fact that this non-crystallographic averaging works, shows that the degree of asymmetry is small, since it does not perturb crystal lattices significantly, nor does the asymmetric feature have enough mass compared to the symmetric elements to bias the particle averaging in EM studies. For MS2 the CP shell follows precisely the organization of a *T* = 3 icosahedral surface lattice except in the one capsomere position taken up by the MP. Such asymmetric capsid features have implications for capsid stability^36^, and therefore play a role in disassembly.

The new cross-linking studies^22^ allowed us to dissect the functions of the MP protein in detail for the first time. Its known functions of sequence-specific RNA binding appear localized to the N-terminal domain, comprising about 40% of the total length, while the C-terminal domain is likely to be the site of the pilin interaction site. Sequence comparisons between phage MPs suggest that these functions are conserved with respect to domains, although there is variability in the RNA-binding domain and extensive conservation of the pilin-binding domain.

PSs must be appropriately presented for CP recognition, and in the examples studied to date this is in the form of an RNA stem-loop. Such structures are transient. Indeed, in isolated oligos their free energy of folding may be positive. They are also in competition with other RNA folds required to promote replication or translation. A question therefore arises as to the mechanism of PS formation. We tested this directly using lead-ion RNA structure probing of CP-free RNAs and the genomic RNA within virions. The data suggest that some PSs are present before encapsidation while others form during the assembly process. Since the RNA exists as an ensemble of folded states, the functional folds of the PSs must flicker in and out of existence until CP binding and initiation of assembly.

## Summary

The assembly of ssRNA viruses, one of the largest classes of viral pathogens that infect organisms in all kingdoms of life, is regulated by multiple, sequence-specific RNA-CP contacts making the process highly co-operative, accurate and efficient. This mechanism is probably also vital for the earliest stages of new infections. These principles have largely been worked out using the RNA phage MS2 which has already provided many firsts in the history of molecular biology, e.g. the first complete genome sequence.^37^ These studies highlight the continuing utility of “model” organisms for understanding the details of the molecular mechanisms underlying complex biological events.

## References

[1] Schneemann A. The structural and functional role of RNA in icosahedral virus assembly. Annu Rev Microbiol 2006; 60:51-67; PMID:16704342; http://dx.doi.org/10.1146/annurev.micro.60.080805.142304 16704342

[2] van der Schoot P, Bruinsma R. Electrostatics and the assembly of an RNA virus. Phys Rev E Stat Nonlin Soft Matter Phys 2005; 71:061928; PMID:16089786; http://dx.doi.org/10.1103/PhysRevE.71.061928 16089786

[3] Belyi VA, Muthukumar M Electrostatic origin of the genome packing in viruses. Proc Natl Acad Sci 2006;103:17174–8; PMID:17090672; http://dx.doi.org/4060587 10.1073/pnas.0608311103 17090672 PMC1859905

[4] Balint R, Cohen SS. The incorporation of radiolabeled polyamines and methionine into turnip yellow mosaic virus in protoplasts from infected plants. Virology 1985; 144:181-93; PMID:4060587; http://dx.doi.org/10.1016/0042-6822(85)90316-2 4060587

[5] Bruinsma RF. Physics of RNA and viral assembly. Eur Phys J E Soft Matter 2006; 19:303-10; PMID:16554977; http://dx.doi.org/10.1140/epje/i2005-10071-1 16554977

[6] Ling CM, Hung PP, Overby LR. Specificity in self-assembly of bacteriophages Qβ and MS2. Biochemistry 1969; 8:4464-9; PMID:4982049; http://dx.doi.org/10.1021/bi00839a036 4982049

[7] Routh A, Domitrovic T, Johnson JE. Host RNAs, including transposons, are encapsidated by a eukaryotic single-stranded RNA virus. Proc Natl Acad Sci USA 2012; 109:1907-12; PMID:22308402; http://dx.doi.org/10.1073/pnas.1116168109 22308402 PMC3277583

[8] Harrison SC, Olson AJ, Schutt CE, Winkler FK, Bricogne G. Tomato bushy stunt virus at 2.9 Å resolution. Nature 1978; 276:368-73; PMID:19711552; http://dx.doi.org/10.1038/276368a0 19711552

[9] Borodavka A, Tuma R, Stockley PG. Evidence that viral RNAs have evolved for efficient, two-stage packaging. Proc Natl Acad Sci USA 2012; 109:15769-74; PMID:23019360; http://dx.doi.org/10.1073/pnas.1204357109 23019360 PMC3465389

[10] Borodavka A, Tuma R, Stockley PG. A two-stage mechanism of viral RNA compaction revealed by single molecule fluorescence. RNA Biol 2013; 10:481-9; PMID:23422316; http://dx.doi.org/10.4161/rna.23838 23422316 PMC3710354

[11] Dykeman EC, Stockley PG, Twarock R. Solving a Levinthal's paradox for virus assembly identifies a unique antiviral strategy. Proc Natl Acad Sci USA 2014; 111:5361-6; PMID:24706827; http://dx.doi.org/10.1073/pnas.1319479111 24706827 PMC3986145

[12] Patel N, Dykeman EC, Coutts RHA, Lomonossoff GP, Rowlands DJ, Phillips SEV, Ranson N, Twarock R, Tuma R, Stockley PG. Revealing the density of encoded functions in a viral RNA. Proc Natl Acad Sci USA 2015; 112:2227-32; PMID:25646435; http://dx.doi.org/10.1073/pnas.1420812112 25646435 PMC4343168

[13] Stockley PG, Rolfsson O, Thompson GS, Basnak G, Francese S, Stonehouse NJ, Homans SW, Ashcroft AE. A simple, RNA-mediated allosteric switch controls the pathway to formation of a *T* = 3 viral capsid. J Mol Biol 2007; 369:541-52; PMID:17434527; http://dx.doi.org/10.1016/j.jmb.2007.03.020 17434527 PMC7612263

[14] Dykeman EC, Stockley PG, Twarock R. Dynamic allostery controls coat protein conformer switching during MS2 phage assembly. J Mol Biol 2010; 395:916-23; PMID:19913554; http://dx.doi.org/10.1016/j.jmb.2009.11.016 19913554

[15] Basnak G, Morton VL, Rolfsson O, Stonehouse NJ, Ashcroft AE, Stockley PG. Viral genomic single-stranded RNA directs the pathway toward a T = 3 capsid. J Mol Biol 2010; 395:924-36; PMID:19913556; http://dx.doi.org/10.1016/j.jmb.2009.11.018 19913556 PMC4785722

[16] Morton VL, Dykeman EC, Stonehouse NJ, Ashcroft AE, Twarock R, Stockley PG. The impact of viral RNA on assembly pathway selection. J Mol Biol 2010; 401:298-308; PMID:20621589; http://dx.doi.org/10.1016/j.jmb.2010.05.059 20621589

[17] Knapman TW, Morton VL, Stonehouse NJ, Stockley PG, Ashcroft AE. Determining the topology of virus assembly intermediates using ion mobility spectrometry–mass spectrometry. Rapid Communications in Mass Spectrometry 2010; 24:3033-42; PMID:20872636; http://dx.doi.org/10.1002/rcm.4732 20872636 PMC4789508

[18] Bunka DHJ, Lane SW, Lane CL, Dykeman EC, Ford RJ, Barker AM, Twarock R, Phillips SEV, Stockley PG. Degenerate RNA Packaging Signals in the Genome of Satellite Tobacco Necrosis Virus: Implications for the Assembly of aT = 1 Capsid. J Mol Biol 2011; 413:51-65; PMID:21839093; http://dx.doi.org/10.1016/j.jmb.2011.07.062 21839093

[19] Ford RJ, Barker AM, Bakker SE, Coutts RH, Ranson NA, Phillips SE, Pearson AR, Stockley PG Sequence-specific, RNA-protein interactions overcome electrostatic barriers preventing assembly of satellite tobacco necrosis virus coat protein. J Mol Biol 2013; 425:1050–64; PMID:23318955; http://dx.doi.org/9180688 10.1016/j.jmb.2013.01.004 23318955 PMC3593212

[20] Valegård K, Liljas L, Fridborg K, Unge T The three-dimensional structure of the bacterial virus MS2. Nature 1990; 345:36-41; http://dx.doi.org/10.1038/345036a0 2330049

[21] Peabody DS. Role of the coat protein-RNA interaction in the life cycle of bacteriophage MS2. Mol Gen Genet 1997; 254:358-64; PMID:9180688; http://dx.doi.org/10.1007/s004380050427 9180688

[22] Rolfsson O, Middleton S, Manfield IW, White SJ, Fan B, Vaughan R, Ranson NA, Dykeman EC, Twarock R, Ford J, Cheng Kao C, Stockley PG Direct Evidence for Packaging Signal-mediated Assembly of Bacteriophage MS2. J Mol Biol 2015; 428(2 Pt B):431–48; PMID:26608810; http://dx.doi.org/9808042 10.1016/j.jmb.2015.11.014 26608810 PMC4751978

[23] Valegård K, Murray JB, Stockley PG, Stonehouse NJ, Liljas L Crystal structure of an RNA bacteriophage coat protein-operator complex. Nature 1994; 371:623–6; PMID:7523953; http://dx.doi.org/9808042 10.1038/371623a0 7523953

[24] Valegård K, Murray JB, Stonehouse NJ, van den Worm S, Stockley PG, Liljas L The three-dimensional structures of two complexes between recombinant MS2 capsids and RNA operator fragments reveal sequence-specific protein-RNA interactions. J Mol Biol 1997; 270:724–38; PMID:9245600; http://dx.doi.org/9808042 10.1006/jmbi.1997.1144 9245600

[25] Rowsell S, Stonehouse NJ, Convery MA, Adams CJ, Ellington AD, Hirao I, Peabody DS, Stockley PG, Phillips SE. Crystal structures of a series of RNA aptamers complexed to the same protein target. Nat Struct Biol 1998; 5:970-5; PMID:9808042; http://dx.doi.org/10.1038/2946 9808042

[26] Convery MA, Rowsell S, Stonehouse NJ, Ellington AD, Hirao I, Murray JB, Peabody DS, Phillips SE, Stockley PG. Crystal structure of an RNA aptamer-protein complex at 2.8 A resolution. Nat Struct Biol 1998; 5:133-9; PMID:9461079; http://dx.doi.org/10.1038/nsb0298-133 9461079

[27] Dykeman EC, Stockley PG, Twarock R Building a viral capsid in the presence of genomic RNA. Phys Rev E Stat Nonlin Soft Matter Phys 2013; 87(2):022717; PMID:23496558; http://dx.doi.org/24036424 10.1103/PhysRevE.87.022717 23496558

[28] Ni P, Vaughan RC, Tragesser B, Hoover H, Kao CC. The Plant Host Can Affect the Encapsidation of Brome Mosaic Virus (BMV) RNA: BMV Virions Are Surprisingly Heterogeneous. J Mol Biol 2014; 426:1061-76; PMID:24036424; http://dx.doi.org/10.1016/j.jmb.2013.09.007 24036424 PMC3944473

[29] Vaughan R, Tragesser B, Ni P, Ma X, Dragnea B, Kao CC. The tripartite virions of the brome mosaic virus have distinct physical properties that affect the timing of the infection process. J Virol 2014; 88:6483-91; PMID:24672042; http://dx.doi.org/10.1128/JVI.00377-14 24672042 PMC4093861

[30] Yi G, Letteney E, Kim C-H, Kao CC. Brome mosaic virus capsid protein regulates accumulation of viral replication proteins by binding to the replicase assembly RNA element. RNA 2009; 15:615-26; PMID:19237464; http://dx.doi.org/10.1261/rna.1375509 19237464 PMC2661835

[31] Dykeman EC, Stockley PG, Twarock R. Packaging signals in two single-stranded RNA viruses imply a conserved assembly mechanism and geometry of the packaged genome. J Mol Biol 2013; 425:3235-49; PMID:23763992; http://dx.doi.org/10.1016/j.jmb.2013.06.005 23763992

[32] Bleckley S, Schroeder SJ. Incorporating global features of RNA motifs in predictions for an ensemble of secondary structures for encapsidated MS2 bacteriophage RNA. RNA 2012; 18:1309-18; PMID:22645379; http://dx.doi.org/10.1261/rna.032326.112 22645379 PMC3383962

[33] Dent KC, Thompson R, Barker AM, Hiscox JA, Barr JN, Stockley PG, Ranson NA. The Asymmetric Structure of an Icosahedral Virus Bound to Its Receptor Suggests a Mechanism for Genome Release. Structure 2013; 21:1225-34; PMID:23810697; http://dx.doi.org/10.1016/j.str.2013.05.012 23810697 PMC3701328

[34] Geraets JA, Dykeman EC, Stockley PG, Ranson NA, Twarock R. Asymmetric genome organization in an RNA virus revealed via graph-theoretical analysis of tomographic data. PLoS Comput Biol 2015; 11:e1004146; PMID:25793998; http://dx.doi.org/10.1371/journal.pcbi.1004146 25793998 PMC4368512

[35] Dykeman EC, Grayson NE, Toropova K, Ranson NA, Stockley PG, Twarock R. Simple Rules for Efficient Assembly Predict the Layout of a Packaged Viral RNA. J Mol Biol 2011; 408:399-407; PMID:21354423; http://dx.doi.org/10.1016/j.jmb.2011.02.039 21354423

[36] Cermelli P, Indelicato G, Twarock R Nonicosahedral pathways for capsid expansion. Phys Rev E Stat Nonlin Soft Matter Phys. 2013; 88(3):032710. PMID:24125297 http://dx.doi.org/1264203 10.1103/PhysRevE.88.032710 24125297

[37] Fiers W, Contreras R, Duerinck F, Haegeman G, Iserentant D, Merregaert J, Jou WM, Molemans F, Raeymaekers A, Van den Berghe A, et al. Complete nucleotide sequence of bacteriophage MS2 RNA: primary and secondary structure of the replicase gene. Nature 1976; 260:500-7; PMID:1264203; http://dx.doi.org/10.1038/260500a0 1264203

